# Pathogenicity and immunogenicity of gI/gE/TK-gene-deleted *Felid herpesvirus 1* variants in cats

**DOI:** 10.1186/s12985-023-02053-8

**Published:** 2023-05-04

**Authors:** Aoxing Tang, Meng Zhu, Jie Zhu, Da Zhang, Shiqiang Zhu, Xiao Wang, Chunchun Meng, Chuangfeng Li, Guangqing Liu

**Affiliations:** 1 Shanghai Veterinary Research Institute, Chinese Academy of Agricultural Sciences (CAAS), Shanghai, 200241 China; 2 College of Animal Science and Technology, Anhui Agricultural University, Hefei, 230036 China

**Keywords:** *Felid herpesvirus 1*, CRISPR/Cas9, Recombinant virus, Vaccine, Cat

## Abstract

**Background:**

*Felid herpesvirus 1* (FHV-1) is a major pathogenic agent of upper respiratory tract infections and eye damage in felines worldwide. Current FHV-1 vaccines offer limited protection of short duration, and therefore, do not reduce the development of clinical signs or the latency of FHV-1.

**Methods:**

To address these shortcomings, we constructed FHV ∆gIgE-eGFP, FHV ∆TK mCherry, and FHV ∆gIgE/TK eGFP-mCherry deletion mutants (ΔgI/gE, ΔTK, and ΔgIgE/TK, respectively) using the clustered regularly interspaced palindromic repeats (CRISPR)/CRISP-associated protein 9 (Cas9) system (CRISPR/Cas9), which showed safety and immunogenicity in vitro. We evaluated the safety and efficacy of the deletion mutants administered with intranasal (IN) and IN + subcutaneous (SC) vaccination protocols. Cats in the vaccination group were vaccinated twice at a 4-week interval, and all cats were challenged with infection 3 weeks after the last vaccination. The cats were assessed for clinical signs, nasal shedding, and virus-neutralizing antibodies (VN), and with postmortem histological testing.

**Results:**

Vaccination with the gI/gE-deleted and gI/gE/TK-deleted mutants was safe and resulted in significantly lower clinical disease scores, fewer pathological changes, and less nasal virus shedding after infection. All three mutants induced virus-neutralizing antibodies after immunization.

**Conclusions:**

In conclusion, this study demonstrates the advantages of FHV-1 deletion mutants in preventing FHV-1 infection in cats.

**Supplementary Information:**

The online version contains supplementary material available at 10.1186/s12985-023-02053-8.

## Introduction

*Felid herpesvirus 1* (FHV-1) is a member of the genus *Varicellovirus* in the family *Herpesviridae* and subfamily *Alphaherpesvirinae* [1]. FHV-1 infection causes feline viral rhinotracheitis (FVR), which is clinically characterized by upper respiratory infections, and also abortion [2, 3]. It is also an important cause of ocular lesions in cats. FHV-1 mainly infects kittens, but can also infect other felines, including tigers and leopards [4–6]. After infection, viral replication in the acute phase of the disease occurs predominantly in the mucosae of the nasal septum, turbinates, nasopharynx, and tonsils. Other tissues, including the conjunctivae, mandibular lymph nodes, and upper trachea, are also often involved [7]. The incidence rate of the disease after infection in cats is up to 100%, but the fatality rate varies greatly in cats of different ages. Adult cats generally do not die, but the mortality rate in kittens can reach 50%. Infected animals are latently infected for life and repeatedly infected under certain stimuli [8, 9]. Since it was first isolated by Crandell and Maurer in 1958 [2], FHV-1 has spread worldwide and is the most clinically significant pathogen causing respiratory infections in cats.

FHV-1 is a double-stranded linear DNA virus, around 126–134 kbp in size, with an overall G + C content of 45–50% [10, 11]. The genomic organization of FHV-1 strains is similar to that of other varicelloviruses. Basically, the FHV-1 genome consists of two unique segments, the unique long (UL) and unique short (US) regions. The US region of the genome is flanked by a pair of identical but inverted sequences, designated the ‘internal repeat short’ (IRS) and ‘terminal repeat short’ (TRS) regions [12]. The functions of 60 proteins have been annotated, and about 23 virus-specific proteins and immunogenic glycoproteins have been identified [13]. Fifty-six genes have been mapped to the UL region of the FHV-1 genome, mainly encoding the capsid glycoprotein B (gB), gC, gK, gL, gM, gN, the myristylated tegument protein CIRC, ribonucleotide reductase (RR), and thymidine kinase (TK). The US region mainly encodes membrane gD, gE, gG, gI, and protein kinase PK [14–16]. Mijnes et al. used the vaccinia virus vTF7-3 expression system to study the biosynthesis of FHV-1 gE and gI proteins [16]. The gE/gI complex is rapidly transported to the endoplasmic reticulum (ER). gI was mainly found in the ER and cell membrane with immunofluorescence detection. An FHV-1 mutant lacking the gI gene did not encode the mature gE protein. It is speculated that gE remained in the ER, which may be related to the lack of a molecular chaperone, and only when it is combined with gI does it have transport activity [17]. Sussman et al. constructed another recombinant FHV-1 that lacked the C-terminus of the gI gene and the 5′ coding region and the 5′ transcriptional control region of the gE gene, and this recombinant virus replicated slowly. The viral titers of the recombinant FHV-1 were approximately tenfold lower than that of the parent. The deletion of gE from FHV-1 is likely to affect its cell-to-cell transmission. After the nasal or subcutaneous injection of the recombinant virus FHV-1, cats were challenged with virulent FHV-1, and showed only mild clinical symptoms [18]. Lee et al. reported that the virulence of FHV-1 was reduced after the TK gene was knocked out, indicating that TK is associated with the pathogenicity of the virus in cats [19].

Inactivated and live modified vaccines against FHV-1 are commercially available and have been relatively successful in controlling FVR [20]. At present, the FHV-1 vaccines suitable for cats mainly include an inactivated vaccine and a modified attenuated vaccine. Although the attenuated vaccine induces higher antibody levels, it can cause latent FHV-1 infections in vaccinated cats and recurrent disease. An inactivated *Feline calicivirus* (FCV)–*Feline panleulopenia virus* (FPV)–FHV vaccine is currently the most widely used and effective vaccine, but the protection afforded by this trivalent vaccine is generally lowest against the FHV-1 component [14, 20, 21]. Importantly, this commercial vaccine only reduces the clinical signs of the disease, but does not prevent infection, virus shedding, or the establishment of latency or reactivation [2, 9]. To protect cats from FHV-1, the development of safe, effective, and economical vaccines is essential. The research and development of gene deletion/insertion vaccines is key to the resolution of this problem. Attempts have been made to improve FHV-1 vaccines with genetic engineering, and a number of FHV-1 deletion/insertion mutants have been developed, including TK deletion mutants. Some of these have incorporated other deletions, including of the FCV capsid gene [22, 23] and gI–gE genes [18], and a gI insertion [24, 25]. In general, these deletion/insertion mutants are less virulent in cats and offer some protection against FVR, especially when administered via the oronasal route [26]. Although no FHV-1-vectored vaccine has yet progressed to commercial use in cats, these vaccines have some utility as potential marker vaccines.

The clustered regularly interspaced short palindromic repeat (CRISPR)-guided manipulation of herpesvirus genomes has recently been developed, and is an efficient method of genome engineering that is likely to be used increasingly in the future [27]. The CRISP/CRISP-associated protein 9 (Cas9) system is a robust and highly efficient tool for gene editing that precisely manipulates specific genomic loci [28, 29]. Gene editing in recombinants by transfecting the CRISPR/Cas9 system is efficient, convenient, and rapid [30, 31].

In this study, a TK-deletion mutant (∆TK), a gI/gE-deletion mutant (∆gI/gE), and a gIgE/TK-deletion mutant (∆gI/gE/TK) were constructed with CRISPR/Cas9 from FHV-1 strain WX19, which was isolated in Wuxi in 2019 (unpublished). We evaluated the immunogenic effects of these deletion mutants in cats towards developing a new feline herpesvirus vaccine candidate.

## Materials and methods

### Cells and viruses

Crandell Reese feline kidney (CRFK) cells were cultured in Eagle’s minimum essential medium (EMEM, Wisent, China) supplemented with 10% heat-inactivated fetal bovine serum (FBS, Gibco, USA), streptomycin (100 µg/mL), and penicillin (100 IU/mL), and incubated under 5% CO_2_ at 37 °C.

*Feline herpesvirus type 1* strain WX19 (FHV WX19) is an (unpublished) field isolate from a young cat with severe rhinotracheitis. To propagate the virus, CRFK cells were infected with it at multiplicity of infection (MOI) of 0.1, and the cell culture supernatant was harvested for titration when a 90% cytopathic effect (CPE) was observed.

### Western blotting

Protein samples were separated on 12% gels and transferred to nitrocellulose membranes (GE Healthcare) with a semidry transfer cell (Bio-Rad Laboratories). The membranes were blocked with 5% nonfat milk in Tris-buffered saline (TBS)–Tween buffer (150 mM NaCl, 20 mM Tris, and 0.05% Tween 20, pH 7.3) for 2 h at room temperature, and then incubated with the following antibodies: anti-green fluorescent protein (GFP) mouse Mab (1:1000; Abcam), anti-mCHERRY mouse Mab (1:1000; Abclonal), anti-gI mouse Mab (1:1000; produced in-house), anti-gE mouse Mab (1:1000; produced in-house), anti-gB rabbit Mab (1:1000; produced in-house), anti-TK rabbit Mab (1:1000; produced in-house), and anti-actin mouse Mab (1:2000; CW). After the membranes were washed five times for 10 min each, they were incubated with a goat anti-mouse IgG secondary antibody conjugated with horseradish peroxidase (1:10,000; Sigma) for 1 h at room temperature. The blots were analyzed with enhanced chemiluminescence (Thermo Fisher Scientific) using an automatic chemiluminescence imager (Tanon).

### Construction of recombinant transfer vectors and single guide RNA (sgRNA) plasmids

The sgRNAs were designed according to http//crispr.mit.edu and targeted the, gI-, gE-and TK-encoding open reading frames (ORFs). The sequences of the sgRNAs are listed in Table 1. The lentiCRISPR v2 plasmid (Addgene) was digested with *Bsm*BI. Primers (Table 1) were designed to amplify the gI/gE and TK homologous arms of FHV strain WX19 with PCR.

**Table 1:** Primer sequences used in this study

Name	Sequences(5′–3′)	Application
TK L arm	F: *AAGCTTGCATGCCTGCAGGTCGAC*GGTTAACGGACGATCTGTGAT	TK L arm amplification
	R: *CCCCGTAATTGATTACTATTAATAACTA*CGTCTGATCTGTGTATGAT	
TK R arm	F: *TAAAGCATTTTTTTCACTGC*ACATTAGTGGTGTTCCCT	TK R arm amplification
	R: *TACCCGGGGATCCTCTAGAGA*GCATTCCATCGGCCAGTAATGTATTAG	
gI/gE L arm	F: *AAGCTTGCATGCCTGCAGGTCGAC*GTGGTGGTGTGTGGCATATTA	gI L arm amplification
	R: *GATTACTATTAATAACTA*CTCCAACCTATTTTATGATGG	
gI/gE R arm	F: *ATTGTAAGCGTTAATATTTTG*CTACTTTGAACGTATCGCATC	gE R arm amplification
	R: *TACCCGGGGATCCTCTAGAGA*GCGGCAGCCGCGGTTTATCTA	
GFP	F: *CCATCATAAAATAGGTTGGAG*TCCTGCGTTATCCCCTGATTC	GFP amplification
	R: *GATGCGATACGTTCAAAGTAG*CAAAATATTAACGCTTACAAT	
mCherry	F: *GTTTCATCATACACAGATCAGACG*TAGTTATTAATAGTAATCAATTACGGGG	mCherry amplification
	R: *AGGGAACACCACTAATGT*GCAGTGAAAAAAATGCTTTATTTGTG	
sgRNA-TK1	F: CACCGAGACTACGCCAGTATAGCAT	TK sgRNA cloning
	R: AAACATGCTATACTGGCGTAGTCTC	
sgRNA-TK2	F: CACCGGGCGGCGGCACATTCATCAG	TK sgRNA cloning
	R: AAACCTGATGAATGTGCCGCCGCCC	
sgRNA-gI/gE1	F: CACCGACACTCAAAAGAGTGAACGT	gI sgRNA cloning
	R: AAACACGTTCACTCTTTTGAGTGTC	
sgRNA-gI/gE2	F: CACCGGTATGTTTCGTGGTGTCTCCG	gE sgRNA cloning
	R: AAACCGGAGACACCACGAAACATACC	
TK-check	F: CATACACAGATCAGACG	Recombinant virus amplification
	R: AGGGAACACCACTAATGT	
gI/gE-check	F: CCATCATAAAATAGGTTGGAG	
	R: GATGCGATACGTTCAAAGTAG	

Bases in italics are the homologous arm for recombination

To replace the gI/E genes of FHV WX19 with the enhanced GFP (eGFP) gene by homologous recombination, we first constructed an expression cassette with the left arm (Larm1) amplified from the FHV WX19 genomic DNA, the eGFP ORF flanked by the *Cytomegalovirus* (CMV) early gene promoter and a polyA signal amplified from peGFP-C3 (Clontech), and the right arm (Rarm1) amplified from the FHV WX19 genomic DNA. The three fragments, Larm1, eGFP, and Rarm1, were joined together by homologous recombination. The final fragment was cloned into pMD-19T (TakaRa) after sequence verification, and the product was designated pT–eGFP. The mCherry-encoding sequence was amplified from pmCherry-C1 (Clontech) and cloned into pMD-19T in the same way, and the product was designated pT–mCherry. In this study, PCR primer synthesis and DNA sequencing were performed by TsingKe Biotech Co. Ltd (Beijing, China).

### Generation of recombinant viruses

To generate the recombinant FHVs (FHV ∆TK mCherry or FHV ∆gIgE eGFP), CRFK cells were cotransfected with 1 µg of Cas9 plasmid sgRNA-gI/gE or sgRNA-TK, respectively, and 2 µg of plasmid pT–eGFP or pT–mCherry, respectively, using Lipofectamine 3000 (Thermo Fisher Scientific) for 18 h. After cotransfection, the cells were infected with FHV strain WX19 at an MOI of 0.1. The cells were maintained in an incubator under 5% CO_2_ at 37 °C until a 90% CPE was observed, and were then collected and subjected to three freeze–thaw cycles. The recombinant FHV was purified from the cell lysates with plaque purification in CRFK cells overlain with 1% low-melting-point agarose and 1% FBS in EMEM. After seven rounds of purification, the plaques were counted under fluorescent microscopy. The recombinant FHV ∆gIgE/TK eGFP-mCherry was constructed in the same way from purified FHV ∆TK mCherry and FHV ∆gIgE eGFP.

### One-step growth curves

CRFK cells were seeded in 96-well plates at 10^5^ cells per well in 1 mL of EMEM 1 day before viral infection. The virus (MOI 1) was allowed to adsorb to the cells at 37 °C for 1 h, the inoculum was removed, and the cells were washed three times with 0.01 M phosphate-buffered saline (PBS; pH 7.2). The cells were maintained in EMEM containing 1% FBS under 5% CO_2_ at 37 °C in an incubator. At the indicated intervals, the cell supernatants were collected for virus titration.

### Viral challenge of cats

Sixteen 3-month-old, prescreened FHV-1-seronegative cats were randomly divided into four groups of four cats each. Three groups of cats were intranasally (IN) immunized with 1 mL of FHV ∆TK mCherry, FHV ∆gIgE eGFP, or FHV ∆gIgE/TK eGFP–mCherry (10^6^ TCID_50_/100 µL). The cats were boosted IN and subcutaneously (SC) with 1 mL of recombinant FHV (10^6^ TCID_50_/100 µL) at 28 days postimmunization (dpi). The cats in the fourth group were not immunized, as the control. All groups were challenged IN with virulent FHV strain WX19 (1 × 10^5^ TCID_50_/100 µL) 3 weeks after the booster vaccination (49 dpi). The clinical signs were recorded daily for up to 63 days. The scoring method used to quantify the clinical symptoms of FHV-1-related disease has been described in a previous study ([26]; see Table 1). Nasal secretions were collected with Dacron swabs every 2 days after the first immunization. Blood samples were collected separately from each cat before vaccination every 7 days. The sera were stored at − 20 °C before antibody testing. The cats were observed daily for 2 months. All the animal experiments were approved by and performed in compliance with the institutional animal care and use guidelines of Shanghai Veterinary Research Institute, Chinese Academy of Agricultural Sciences.

### Virus isolation

Nasal swab eluates were filter-sterilized with Millex® syringe filter units (Millipore, Bedford, MA, USA), with an average pore diameter of 0.45 µm. For the standard virus isolation assay, 2.5 × 10^4^ CRFK cells and 50 µL of filtrate were added to duplicate wells of a 96-well microtiter plate. The plates were incubated for 3 days at 37 °C under 5% CO_2_ and the presence or absence of a characteristic CPE was evaluated to determine whether the samples were positive or negative.

### Virus-neutralizing antibody testing

The sera were heat-inactivated at 56 °C for 30 min and then a series of two-fold dilutions was prepared. FHV WX19 (200 TCID_50_/100 µL) was mixed with the samples with an equal volume. After incubation at 37 °C for 1 h, 1 × 10^4^ CRFK cells were added to each well and incubated for 3 days at 37 °C under 5% CO_2_. Each sample was tested in duplicate.

### Gross pathology and histological examination

Samples of nasal mucosa, tonsil, trachea, lung, retropharyngeal lymph node, heart, brain, liver, kidney, and spleen were collected, fixed in 10% buffered formalin, embedded in paraffin, sectioned, and routinely stained with hematoxylin and eosin. All sections were examined by a pathologist to evaluate any pathological changes.

### Statistical analysis

Before analysis of the data, the normality of repeated measures and the homogeneity of variance were tested with the Shapiro–Wilk test and Levene’s test, respectively (GraphPad Prism Software v6). For VN antibody titers, the data was log transformed to normalize the data, Student’s *t* test and one-Way ANOV test was used for analysis (GraphPad Prism Software v6). *P* < 0.05 was deemed to indicate significant differences, and *P* < 0.01 was deemed to indicate highly significant differences.

## Results

### Generation of recombinant viruses

**Fig. 1:**
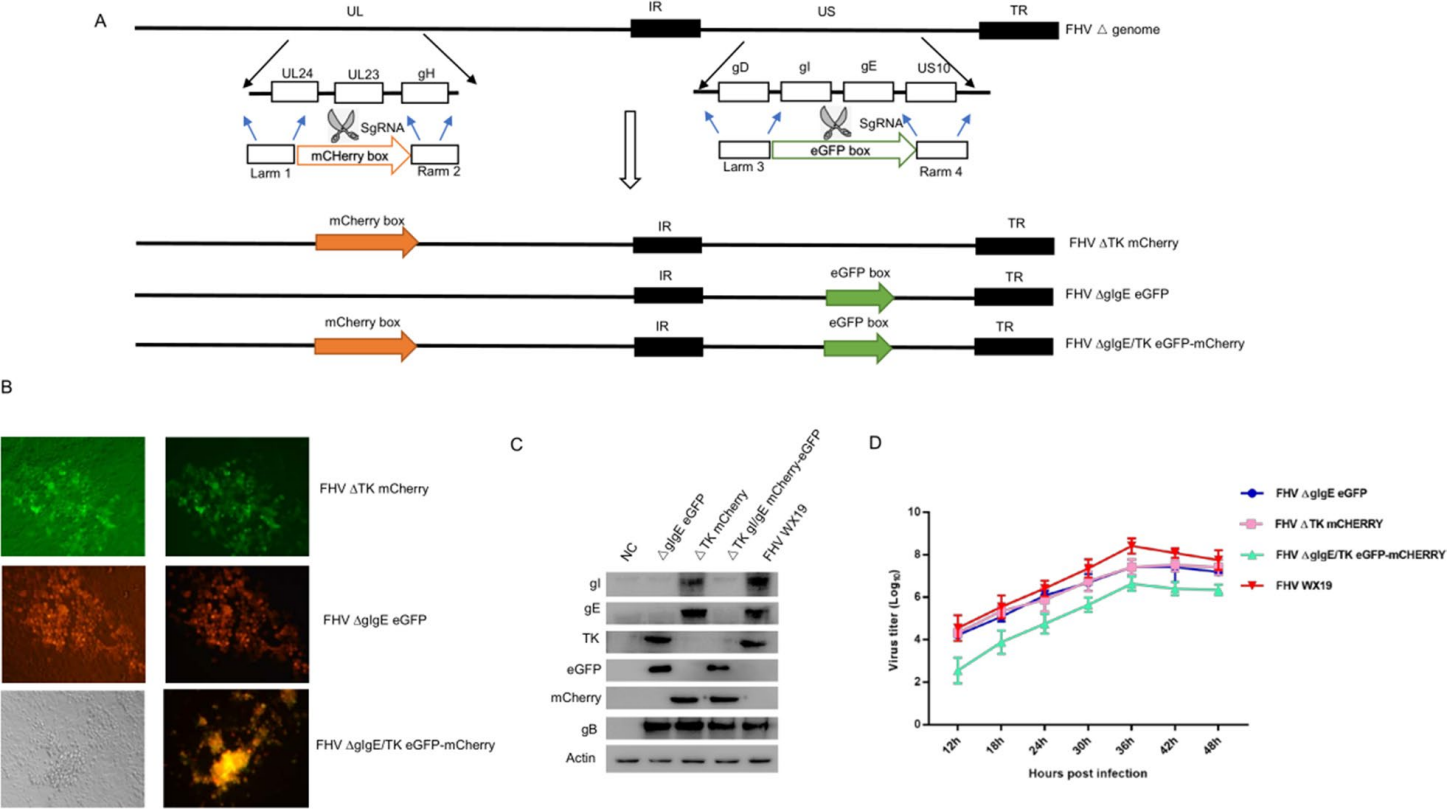
Construction of recombinant FHV WX19 with deletion of the gI, gE, and TK genes. **A** Diagram of the FHV TK, gE, and gI gene deletions. The transfer plasmids pT–eGFP and pT–mCherry were constructed for homologous recombination with the cotransfection of sgRNAs, followed by the infection of cells with FHV strain WX19. **B** FHV ∆gIgE eGFP, FHV ∆TK mCherry and FHV ∆gIgE/TK eGFP–mCherry show light field and and florescence dark field, respectively. **C** CRFK cells were infected (MOI = 0.1) with FHV WX19 (line 5), FHV ∆gIgE eGFP (line 2), FHV ∆TK mCherry (line 3), FHV ∆gIgE/TK eGFP–mCherry (lane 4), or mock infected (lane 1). At 48 h postinfection (hpi), the levels of the gI, gE, TK, and gB proteins were determined with western blotting. **D** One-step growth curves of the recombinant viruses in CRFK cells

CRFK cells were cotransfected with the sgRNA-gI/gE, sgRNA-TK, pT-eGFP, or pT-mCherry plasmid for 18 h and then infected with FHV wild-type strain WX19. The mutants were collected and purified with seven rounds of plaque purification (Fig. 1B). The FHV mutants expressing eGFP, mCHERRY, or both EGFP and mCHERRY were purified and identified as FHV ∆gIgE eGFP, FHV ∆TK mCherry, or FHV ∆gIgE/TK eGFP–mCherry, respectively (Fig. 1). PCR using primers gI/gE-check and TK-check revealed a clear band after electrophoresis, consistent with the expected size of gI/gE and TK deletion and replacement with eGFP and mCherry (Additional file 1: Fig. S1). One-step growth curves showed that the in vitro growth of FHV ∆gIgE eGFP and FHV ∆TK mCherry was similar to that of the FHV wild-type strain WX19, whereas that of FHV ∆gIgE/TK eGFP-mCherry was attenuated compared with that of parental strain FHV WX19 (Fig. 1D).

### gI/gE- and gI/gE/TK-deleted recombinant viruses are safe

**Fig. 2:**
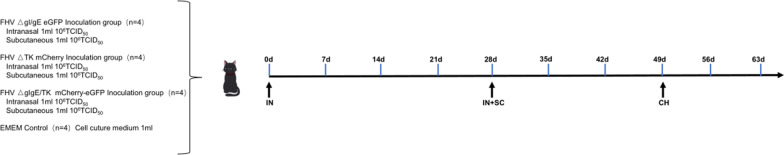
Timeline and experimental design of the study. The cats in the ∆gIgE, ΔTK, and ΔgIgE/TK groups were intranasally inoculated with the FHV ΔgIgE eGFP, FHV ΔTK mCherry, and FHV ΔgIgE/TK eGFP-mCherry mutants, respectively, on days 0 and 28, followed by subcutaneous inoculation with the respective mutant on day 28. The control group was not inoculated. All four groups were challenged intranasally with virulent FHV WX19 (CH) on day 49

**Fig. 3:**
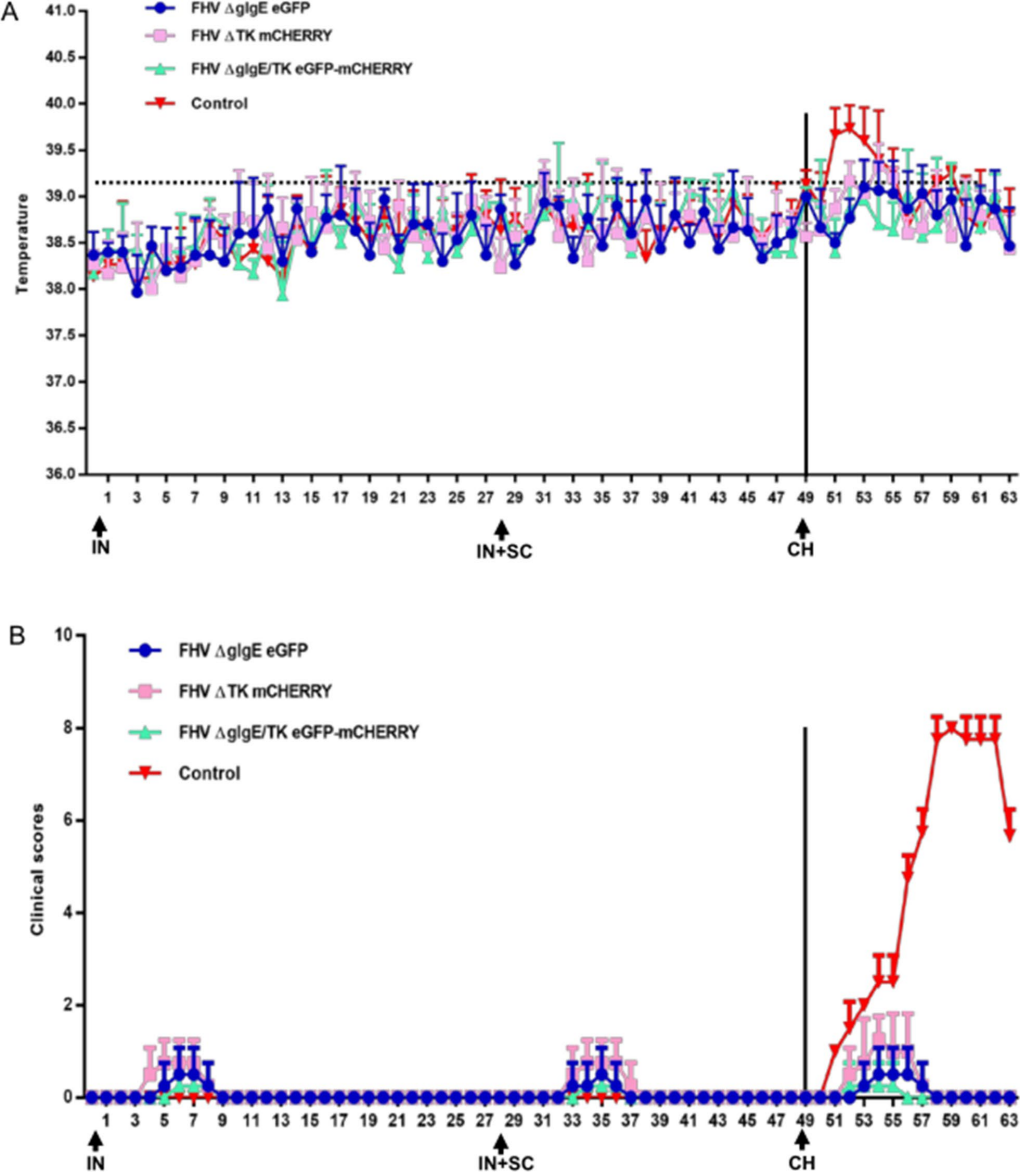
Clinical assessment. **A** Intra-ear temperatures of cats after immunization and challenge. Intra-ear temperatures were measured with a digital thermometer on the indicated days. Mean temperatures and standard deviations (SD) for each treatment group (n = 4) are shown. **B** Clinical scores for cats after immunization and challenge. Symptoms were recorded daily by a blinded observer. Mean temperatures and standard deviations (SD) for each treatment group (n = 4) are shown

Before vaccination, the clinical scores were zero for all groups. After the first IN immunization, mild sneezing was observed in 8 of 16 kittens: two kittens in the ΔgI/gE group, two in the ΔgI/gE/TK group, and four in the ΔTK group. After the second IN immunization, the symptoms were the same as after the first, except that the duration of symptoms was shorter. The inner-ear temperatures were measured in all kittens and none of the kittens showed increased body temperature before the challenge. The kittens in all groups were IN challenged at 49 dpi with FHV WX19 (1 × 10^5^ TCID_50_/mL). All the kittens in the control group showed typical clinical signs (sneezing, watery eyes, loss of appetite, and conjunctivitis) and high fever (> 39.2 °C) (Fig. 2A,B). Two kittens in the ΔgI/gE vaccine group showed sneezing on day 4 after challenge. All the kittens in the ΔTK vaccine group showed clinical signs of sneezing and watery eyes only, with no fever. Only one cat in the ΔgI/gE/TK vaccine group showed sneezing after the challenge (Fig. 3; Table 2).

**Table 2:** Components of the clinical score

Category	Signs	Score
Conjunctivitis	Normal	0
	Mild hyperemia	1
	Moderate to severe hyperemia	2
	Moderate to severe hyperemia, with chemosis	3
Blepharospasm	Normal	0
	< 25% of eye closed	1
	25–50% of eye closed	2
	50–75% of eye closed	3
	75–100% of eye closed	4
Ocular discharge	75–100% of eye closed	0
	75–100% of eye closed	1
	75–100% of eye closed	2
	75–100% of eye closed	3
Sneezing	Normal	0
	Observed	1
Nasal discharge	Normal	0
	Minor, serous, occasional with blood	1
	Minor to moderate, mucoid or bloody	2
	Marked mucopurulent	3
Nasal congestion	Normal	0
	Mild	1
	Moderate	2
	Severe, with open mouth breathing	3
Coughing	Normal	0
	Coughing noted	1

### Protective effects of gI/gE- and gI/gE/TK-deleted recombinant viruses in cats

**Fig. 4:**
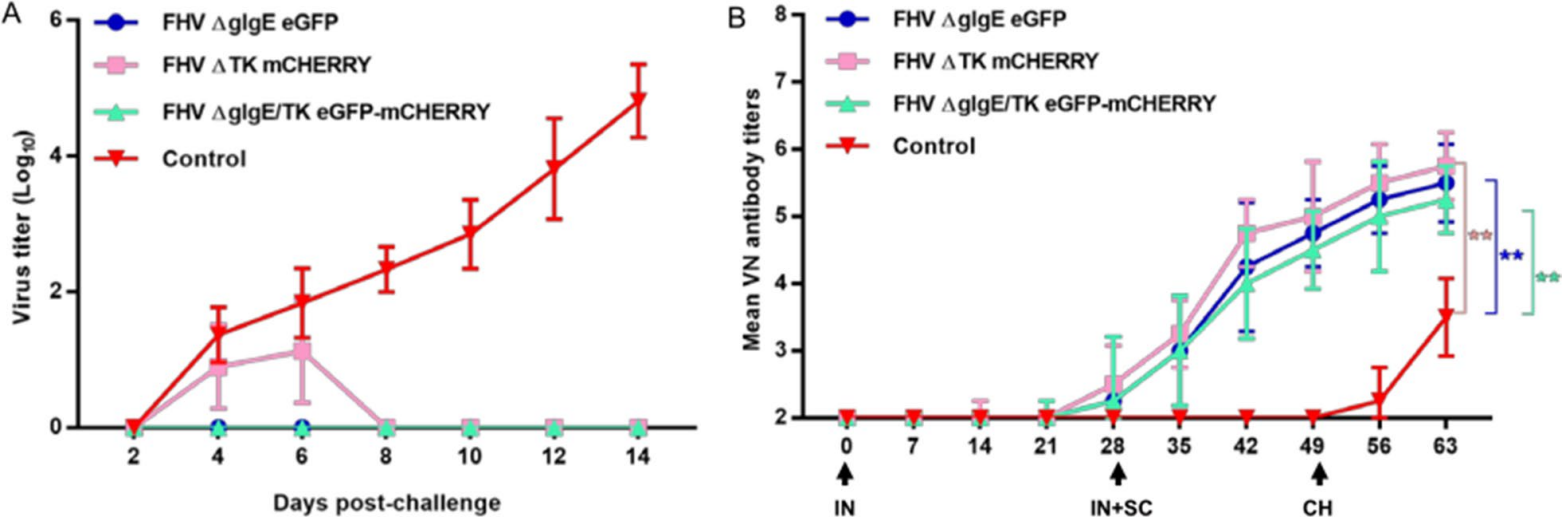
Protective efficacy of gI/gE- and gI/gE/TK-deleted recombinant viruses against FHV WX19 challenge in kittens. **A** Nasal swab samples by cats after FHV WX19 challenge. **B** Neutralizing antibody levels in cats after immunization and challenge. Virus-neutralizing antibodies against FHV: the reciprocal of the highest serum dilution inhibiting CPE formation in 50% of wells infected with 100 TCID_50_ FHV WX19. FHV-1 neutralizing antibody titers of control group (n = 4) or the recombinant virus groups (n = 4) (mean + standard deviation). Asterisks indicate statistically significant differences between the control group and the groups of three mutants: **P < 0.01

The virus-neutralizing antibody titers in the kittens after vaccination and challenge are shown in Fig. 4. On day 28, 4 weeks after the first IN vaccination, the virus-neutralizing antibody titers were slightly elevated in the three test groups. On day 49, 21 days after the second SC and IN inoculations, the virus-neutralizing antibody titers in the three test groups had increased significantly. By day 63, 14 days after infection with the wild-type strain, the antibody titers of all three vaccination groups were significantly higher than those in the control group, but were not significantly different from one another.

Nasal swab samples were collected at the end of the challenge period, and the virus was isolated to detect viral shedding. As shown in Fig. 4A, FHV was only detected in the nasal swab samples from the control group and ΔTK group. The titers of FHV excreted in the EMEM-treated control group were higher than those in the ΔTK group. The titers of FHV were consistently higher in the control group, whereas the titers in the ΔTK group peaked at 6 dpi, with an average titer of 10^1.5^ TCID50/mL. The ΔTK test group stopped excreting FHV by 8 dpi. The ΔgI/gE group and ΔgI/gE/TK group shed no virus.

### Inoculation with ΔgI/gE and ΔgI/gE/TK recombinants reduced the lesions in the lungs and turbinates relative to those in the control cats

**Fig. 5:**
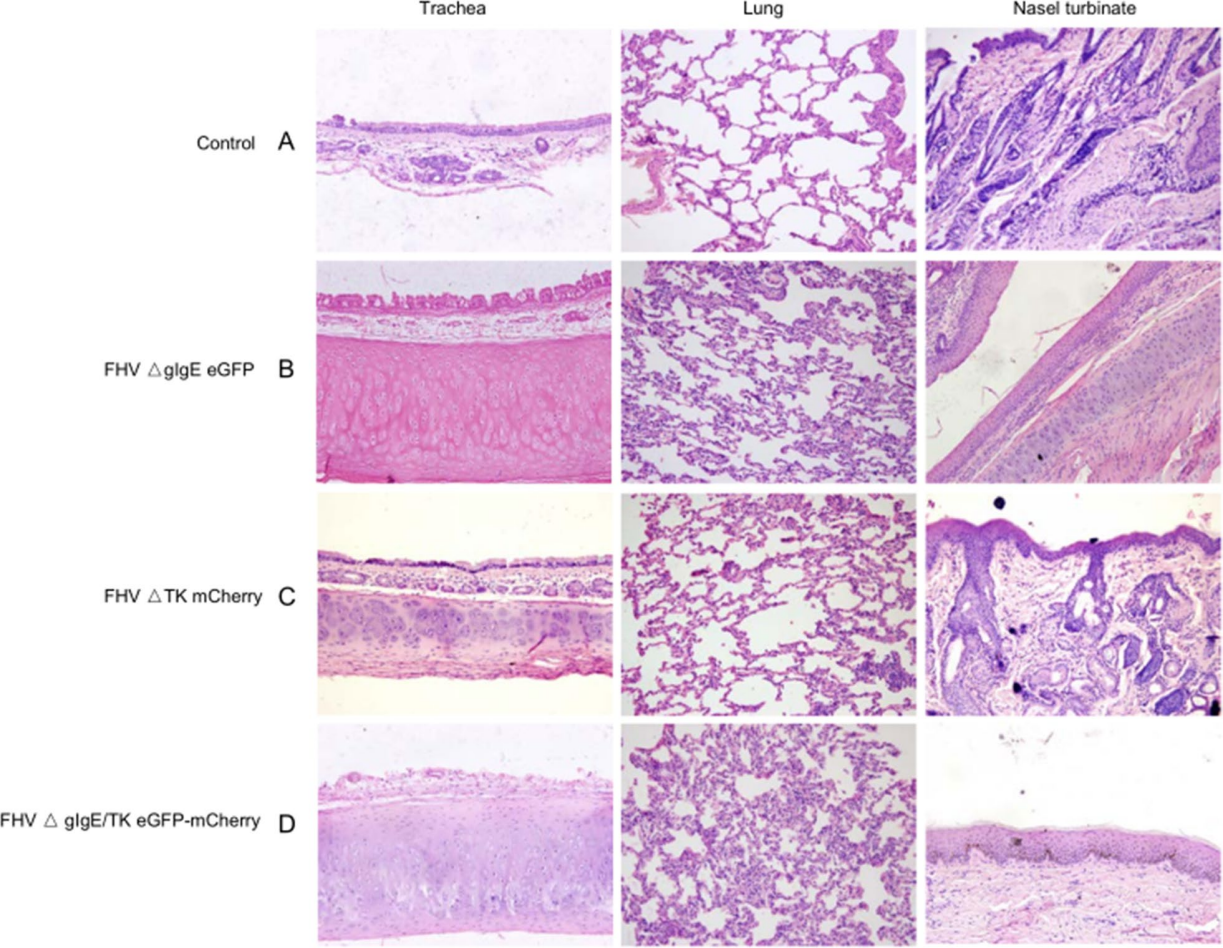
Histological changes in trachea, lungs, and nasal turbinates at autopsy. Representative images from the control group (**A**); ΔgIgE group (**B**); ΔTK group (**C**); and ΔgIgE/TK group (**D**). Scale bar is 100 µm

Inoculation with the gI/gE- and gI/gE/TK-deleted recombinant virus resulted in fewer lesions in the trachea, lungs, and nasal turbinates than in the control cats at the postmortem examination 2 weeks after infection. The lesions in all the cats were limited to the trachea, lungs, and nasal turbinates, and microscopic images are shown in Fig. 5. All cats in the control group had mild interstitial pneumonia with congestion in the lungs, diffuse lung lesions, alveolar edema, and interstitial macrophage and lymphocyte infiltration. All four cats had chronic lymphoplasmacytic rhinitis. In contrast, the histological changes in the ΔgI/gE and ΔgI/gE/TK groups were much milder, particularly in the nasal turbinates. Some of the cats in the ΔgI/gE/TK group had some intra-alveolar edematous fluid and a few intra-alveolar macrophages in the lungs, whereas most cats had normal nasal mucosae (Fig. 5) and no obvious changes in the lungs. In the TK group, all four cats had mild interstitial pneumonia with intra-alveolar macrophage infiltration, and three of the four cats had mild rhinitis. No microscopic damage was detected in the other organs or tissues examined in this study in any group.

## Discussion

FHV-1 has been a subject of research into feline vector vaccines for the last century [22, 32]. In recent years, the CRISPR/Cas9 technology has been widely used for virus editing and vaccine development [33, 34]. Safe gene-deficient viruses can be generated with the CRISPR/Cas9 technology to stimulate a more comprehensive immune response with the combination of both SC and mucosal routes of administration. Gene-deleted viruses have been used extensively to vaccinate against *Pseudorabies virus* [35–37], *Bovine herpesvirus 1*, and *Equid herpesvirus 1* [38, 39].

FHV-1 is currently diagnosed in approximately 50–75% of feline viral upper respiratory infections and has become an important source of infection in cats [2, 14]. Traditional vaccines for FHV-1 are not very effective, reducing the severity of the clinical signs, but conferring only limited or short-term protection, and do not prevent FHV-1 infection [40]. *Herpesviridae* genomes are ideal viral vectors, with the advantages of both the safety of an inactivated vaccine and the immunological efficacy and low cost of a live vaccine, while inducing broad humoral and cellular immunity in the treated organism [34, 41, 42]. The virulence of FHV-1 is significantly reduced by the deletion of the appropriate virulence genes, gI, gE, PK, or TK [15, 43]. In a previous study, C7301d/TK was constructed by deleting the TK gene, and significant protection was observed after challenge with the parental FHV-1 strain C7301, which was inoculated multiple times with recombinant C7301d/TK by different routes. However, high titers of the challenge virus were excreted from the target organs [38]. The recombinant strain FHVβ-galgIgE was constructed by deleting the FHV-1 gI and gE genes. The IN and SC inoculation routes were compared and the ability of the recombinant to confer protective immunity and prevent viral shedding after challenge was assessed. Cats inoculated with high doses of strain FHVβ-galgIgE in nasal drops showed only mild clinical signs and developed strong immunity after challenge with the wild-type virus [18].

In the present study, a TK-deletion mutant (ΔTK), a gIgE-deletion mutant (ΔgI/gE), and a gI/gE/TK-deletion mutant (ΔgI/gE/TK) were prepared with the CRISPR/Cas9 technology by deleting the gI, gE, and/or TK genes from FHV-1 strain WX19. Studies have shown that simultaneous vaccination via the SC and IN routes provides better protection against the clinical signs of FHV-1 infection and viral shedding than are observed in kittens 1 week after SC vaccination [26]. In the present study, the ΔgI/gE and ΔgI/gE/TK groups showed good safety profiles after two mucosal and one SC booster immunization. Only 50% of the ΔgI/gE and ΔgI/gE/TK groups showed mild clinical signs before challenge, whereas 100% of the test cats in the ∆TK group showed clinical signs. This indicates that the ΔgI/gE and ΔgI/gE/TK recombinants were safer than the ∆TK recombinant. In contrast, only 2/4 kittens in the ΔgI/gE group showed sneezing after challenge, whereas 4/4 kittens in the ΔTK group showed sneezing and watery eyes. However, only 1/4 kittens in the ΔgI/gE/TK vaccine group showed sneezing. Moreover, only one cat in the ΔgI/gE/TK group had a fever lasting for 1 day after challenge. In contrast, 2/4 of the cats in the ΔgI/gE group showed occasional fever lasting 2 days after the challenge, although no live virus was isolated from the noses of this group. In contrast, 3/4 of the cats in the ΔTK group had occasional febrile reactions lasting 2–3 days after challenge, and live virus was isolated from the noses of all cats in this group.

In alphaherpesvirus infections, the cell-mediated immune response is more important than humoral immunity [44]. However, the cellular immune response can only be measured with sophisticated laboratory methods [44, 45]. It has been shown that serum antibody testing is not useful in predicting the protection conferred against FHV infection [46] because FHV-1 vaccines act by inducing neutralizing antibodies and cellular immunity [47, 48].

An FHV-1 vaccine administered IN provides early protection by triggering local FHV-1-specific immunoglobulin M (IgM) and IgA and can induce the same or a higher humoral immune response [26, 49]. In a challenge model of FHV-1-infected kittens, the simultaneous IN and SC administration of an FHV-1 vaccine was superior to its SC administration alone. Furthermore, kittens vaccinated simultaneously via the IN and SC routes showed better protection against clinical signs of FHV-1 infection and less viral shedding than those vaccinated via one route only [26]. The mechanisms involved in the synergy of coimmunization are unknown, but this synergy explains the prevention of clinical signs and viral shedding in the test groups after challenge. However, the clinical signs and viral shedding were significantly greater in the ΔTK group than in the ΔgI/gE and ΔgI/gE/TK groups.

In the ΔgI/gE and ΔgI/gE/TK groups, the lack of the circulating memory B-cell subsets that produce antibodies that recognize gI and gE may have affected the complement-dependent and complement-independent virus-neutralizing antibody levels [50]. This also explains why the ΔgI/gE and ΔgI/gE/TK groups had lower virus-neutralizing antibody levels after challenge than the ΔTK group. However, 3/4 of the cats in the TK group displayed fever after challenge, an indicator of an ongoing systemic immune response that is usually associated with the development of virus-neutralizing antibodies after immunization [19, 20, 26]. This suggests that the ΔTK group had relatively high virus-neutralizing antibody levels.

In summary, in this study, we undertook comprehensive safety and efficacy assessments of three test vaccine candidates (gI/gE-, TK-, and gI/gE/TK-deletion mutants of FHV-1) with an immunization program that included IN immunization followed by SC immunization. The parameters evaluated in this study included the safety of the recombinant constructs, their induction of immune responses, and the protection they conferred against challenge with a clinical FHV-1 isolate.

## Conclusions

Our data clearly demonstrate that vaccination with the gI/gE- and gI/gE/TK-deleted mutants reduced the symptoms of clinical disease and viral shedding, and improved the neutralizing antibody levels.

## Supplementary Information

Additional file 1: Figure S1 Construction of recombinant FHV WX19 with deletion of the gI, gE, and TK genes. A: presence of gI/E, fragment in the parenteral FHV by PCR, deletion and replacement of the counterpart by eGFP in the FHV-ΔgI/E- eGFP and FHV ΔgIgE/TK eGFP–mCherry. B: presence of TK, fragment in the parenteral FHV by PCR, deletion and replacement of the counterpart by eGFP in the FHV-ΔTK-mCherry and FHV ΔgIgE/TK eGFP–mCherry.

## Data Availability

The datasets used and analyzed during the current study are available from the corresponding author on reasonable request.

## Abbreviations

FHV-1: *Felid herpesvirus 1*
CRISPR: Lustered regularly interspaced palindromic repeats
Cas9: CRISP-associated protein 9
IN: Intranasal
SC: Subcutaneous
VN: Virus-neutralizing antibodies
FVR: Feline viral rhinotracheitis
UL: Unique long
US: Unique short
IRS: Internal repeat short
TRS: Terminal repeat short
gB: Capsid glycoprotein B
RR: Ribonucleotide reductase
TK: Thymidine kinase
ER: Endoplasmic reticulum
FCV: *Feline calicivirus*
FPV: *Feline panleulopenia virus*
CRFK: Crandell Reese feline kidney
EMEM: Eagle’s minimum essential medium
FBS: Fetal bovine serum
MOI: Multiplicity of infection
CPE: Cytopathic effect
sgRNA: Single guide RNA
