# Supplementary material for: Pathogenicity and immunogenicity of gI/gE/TK-gene-deleted Felid herpesvirus 1 variants in cats
Source: Virol J. 2023 May 4;20:87. doi: 10.1186/s12985-023-02053-8 (PMC10157573; doi:10.1186/s12985-023-02053-8)
Supplement: Supplementary file 1 — Additional file 1: Figure S1 Construction of recombinant FHV WX19 with deletion of the gI, gE, and TK genes. A: presence of gI/E, fragment in the parenteral FHV by PCR, deletion and replacement of the counterpart by eGFP in the FHV-ΔgI/E- eGFP and FHV ΔgIgE/TK eGFP–mCherry. B: presence of TK, fragment in the parenteral FHV by PCR, deletion and replacement of the counterpart by eGFP in the FHV-ΔTK-mCherry and FHV ΔgIgE/TK eGFP–mCherry. [file 12985_2023_2053_MOESM1_ESM.pptx]

## Slide 1
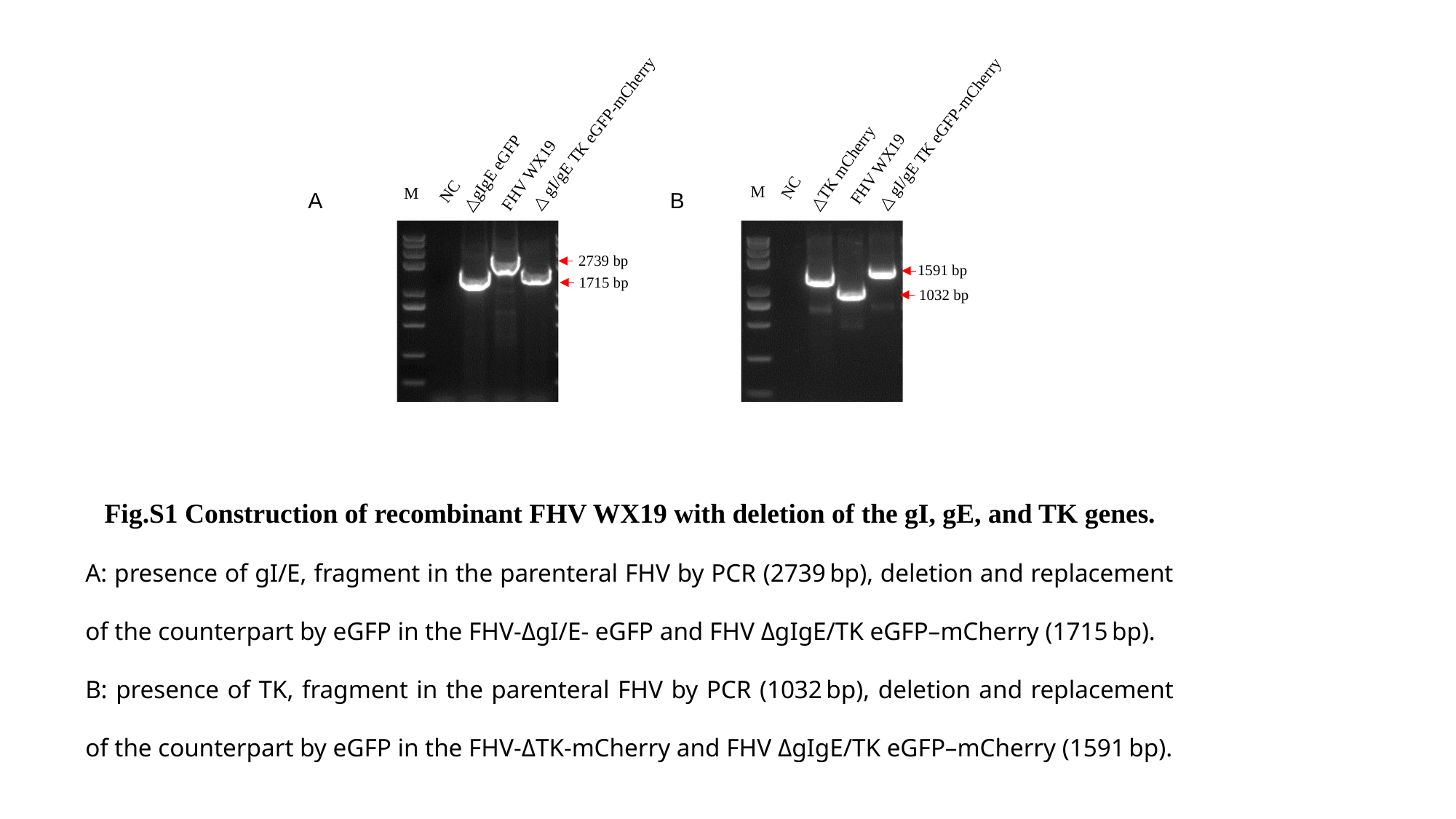

△ gI/gE TK eGFP-mCherry
FHV WX19
△gIgE eGFP
NC
2739 bp
1715 bp
M
△TK mCherry
FHV WX19
NC
1591 bp
△ gI/gE TK eGFP-mCherry
1032 bp
M
A
B
Fig.S1 Construction of recombinant FHV WX19 with deletion of the gI, gE, and TK genes.
A: presence of gI/E, fragment in the parenteral FHV by PCR (2739 bp), deletion and replacement of the counterpart by eGFP in the FHV-ΔgI/E- eGFP and FHV ΔgIgE/TK eGFP–mCherry (1715 bp).
B: presence of TK, fragment in the parenteral FHV by PCR (1032 bp), deletion and replacement of the counterpart by eGFP in the FHV-ΔTK-mCherry and FHV ΔgIgE/TK eGFP–mCherry (1591 bp).
